# Fin Whale (*Balaenoptera physalus*) Mortality along the Italian Coast between 1624 and 2021

**DOI:** 10.3390/ani12223111

**Published:** 2022-11-10

**Authors:** Valerio Manfrini, Nino Pierantonio, Alessandro Giuliani, Federico De Pascalis, Nicola Maio, Annalaura Mancia

**Affiliations:** 1 Independent Researcher, 44022 Comacchio (FE), Italy; 2 Tethys Research Institute, 20121 Milan, Italy; 3 Environment and Health Department, Istituto Superiore di Sanità (National Institute of Health), 00161 Rome, Italy; 4 BIO-AVM, Istituto Superiore per la Protezione e la Ricerca Ambientale (ISPRA), 40064 Ozzano dell’Emilia, Italy; 5 Dipartimento di Biologia, Università degli Studi di Napoli Federico II, 80126 Naples, Italy; 6 Dipartimento Scienze Della Vita e Biotecnologie, Università di Ferrara, 44121 Ferrara, Italy

**Keywords:** fin whale, *Balaenoptera physalus*, strandings, ship strikes, mortality events, anthropogenic threats, Mediterranean Sea

## Abstract

**Simple Summary:**

We present a comprehensive overview of fin whale historical and modern mortality events that occurred between 1624 and 2021 along the Italian coast, highlighting spatial and temporal patterns and, where possible, the proximal causes of mortality. Emerging hot spot analysis shows the spatial and temporal consistency of mortality events along the northern coast of the island of Sardinia, the central coast of Tuscany and the Gulf of Trieste in the northern Adriatic Sea. The coast of Liguria and the northern coast of Tuscany are sporadic hot spots, while the central coast of Italy along the Tyrrhenian Sea as well as the coast of southern Sardinia and northern Sicily have been identified as new hot spots of mortality events for the species. While the analysis of the temporal patterns suggests a steep increase in the number of mortality events starting in the second half of the 1980s, we cannot exclude the possibility that this positive trend is the result of a strong observer bias. Conversely, recent mortality events seem to be consistent in number over the last six decades and subject to year-round seasonality. Our results show that younger and immature individuals are the fin whales most affected by ship strikes. This study supports the implementation of a conservation plan to ensure the survival of the species in the Mediterranean region.

**Abstract:**

The Mediterranean Sea hosts a population of fin whale (*Balaenoptera physalus*), the only species of Mysticete regularly occurring in the basin. Observed and inferred mortality suggests that the population is likely declining. Accordingly, understanding the causes of mortality and assessing the health status is pivotal to the survival of this endangered population. While such studies are inherently difficult for a highly roaming species with a pelagic distribution, mortality events provide the opportunity to investigate biological and epidemiological traits linked to these events, and evaluate the footprint of human activity, especially when long-term data series exist. We present a comprehensive spatial–temporal overview of fin whale mortality events along the Italian coast encompassing four centuries (1624–2021). Time series analysis was used to highlight structural changes in the evolution of mortality through time, while spatial–temporal patterns in the distribution of mortality events were assessed through emerging hot spot analysis methods. Recent mortality events (1964–2021) were further explored to evaluate, where possible, the primary causes of mortality and to identify anthropogenic threats of conservation concerns. This long-term survey offers the basis for an understanding of the health status of this *B. physalus* population and provides much-needed information for developing an effective management and conservation plan for the species in the region.

## 1. Introduction

The Mediterranean Sea is a hot spot of biodiversity and is home to several species of conservation concern [1]. In the basin, species and habitats alike are under ever-increasing pressure derived from human activity with the potential to severely undermine their survival. While it is crucial to reduce the human footprint on the Mediterranean ecosystem and manage threats, conservation efforts are often limited for highly mobile and cryptic species, due to the lack of relevant information on abundance, distribution, and habitat use. This is particularly true for cetaceans for which the collection of baseline information is often hampered by the complex ecological traits of the target species. In this context, historical and modern records of mortality events have proved particularly successful in shedding light on several aspects of cetaceans’ biology and ecology, and supporting informed conservation and management decisions [2,3,4,5,6,7,8]. Post-mortem examinations of both live- and dead-stranded cetaceans could, in fact, enable disease detection in otherwise inaccessible wild populations [9] and allow for health assessments aimed at understanding the diseases, pathogens, and anthropogenic activities afflicting wild cetaceans [10,11,12,13]. Additionally, although the quality of the data derived from post-mortem examinations is highly contingent upon the status of the specimen, and, therefore, often the causes of cetacean mortality remain largely unknown, the analysis of mortality events can lay the basis for developing quantitative frameworks for testing hypotheses regarding the correlates of mortality [14,15,16]. Finally, studies of mortality events can be used to suggest recommendations for managing cetacean carcasses, identifying cost-effective disposal methods that minimise costs, and maximise ecosystem functions and services [17].

The fin whale *Balaenoptera physalus* (Linnaeus, 1758) is the only Mysticete that regularly inhabits the Mediterranean Sea, where two distinct genetic lineages occur: true Mediterranean fin whales that reside year-round in the basin; and northeastern North Atlantic (NENA) fin whales, which enter the basin through the Gibraltar Strait into the western most sector of the Mediterranean Sea [18,19]. Fin whales aggregate during the summer in the feeding grounds of the Pelagos Sanctuary for Mediterranean Marine Mammals [20] and move out to winter-feeding and breeding grounds across the basin [21]. A direct connection between the winter and summer feeding grounds in the Strait of Sicily and the northwestern Mediterranean, respectively, has been recently highlighted [22]. The extent and timing of Mediterranean fin whale movements within the basin are still under debate, with new evidence showing that relatively high numbers of fin whales extend their stay in the northwestern Mediterranean well into the winter months [23].

A region-wide population estimate of fin whales in the Mediterranean was derived from the 2018 ACCOBAMS Survey Initiative, resulting in a corrected estimate of 3280 individuals, all of them sighted in the western Mediterranean Sea [24]. The only earlier effort that is comparable in methods and area with the 2018 monitoring was conducted in 1991 and produced an estimated abundance of 3583 individuals [25], 901 of which were feeding in the Corsican-Ligurian-Provençal basin during the summer months [26]. A comparison of these estimates suggests a putative population decrease of about 10% over the last 27 years [27].

The Mediterranean population of fin whales is currently classified as Endangered according to IUCN Red List Criteria [27]. In addition, the species is listed in Appendix I of CITES (Convention on International Trade in Endangered Species of Wild Fauna and Flora). As a Mediterranean cetacean species, its conservation is also included in several other international conventions: it is listed in Annex II of the SPAMI (Specially Protected Areas of Mediterranean Importance or Barcelona Convention); in Appendix II of the Bern Convention; in Annex IV of the EU Habitats Directive (Directive 43/92/EC); as ‘endangered migratory species’ in Appendix I and II of the Bonn Convention; and as protected species in Annex 1 of the Agreement on the Conservation of Cetaceans of the Black Sea, Mediterranean Sea and contiguous Atlantic Area (ACCOBAMS). In Italy, fin whales are protected, like other cetaceans, by the Law on the Protection of Fauna (Law n. 157 of 11 February 1992).

Cetacean mortality events are relatively common in the Mediterranean Sea, with several cases of unusual die-off events occurring across the region [28,29,30,31] with the potential to afflict the populations and species [32]. Specifically concerning Mediterranean fin whales, evidence suggests that increased mortality is the result of exposure to direct and indirect anthropogenic activities, among which ship strikes are considered the primary cause [33,34,35,36,37,38]. However, recent studies have cautiously highlighted how dolphin morbillivirus (DMV) infections should be regarded as one of the main threats to Mediterranean fin whales [30]. Additionally, exposure to chemical contaminants of different kinds (e.g., macro- and microplastics, POPs, trace metals) [39,40,41,42], pathogens [28,30], acoustic pollution [43] and entanglement in fishing gear [42] may impact the population. Habitat degradation, depletion of fish resources and other climate-induced changes in marine circulation regimes are also suggested as potential threats to the population [44].

In Italy, the first attempts to systematically collect data on cetacean mortality and strandings were made by the World Wildlife Fund through the ‘Cetacean Project’ at the end of the 1970s, followed by the efforts of the Centro Studi Cetacei (CSC), the first Italian national stranding network, which was established in 1985 and started its nationwide activities in 1986 [45]. To date, the activities of CSC are concentrated in certain regions of the Adriatic Sea.

Since 2006, the Interdisciplinary Center for Bioacoustics and Environmental Research (Centro Interdisciplinare di Bioacustica e Ricerche Ambientali, CIBRA) of the University of Pavia and the Museum of Natural History of Milan have added to the fundamental role played by the CSC. They were charged by the Italian Ministry of the Environment and the Protection of the Territory and the Sea (Ministero dell’Ambiente e della Tutela del Territorio e del Mare) to collect cetacean stranding data. As part of this assignment, the Italian stranding network database (Banca dati spiaggiamenti, BDS—http://mammiferimarini.unipv.it/index_en.php (accessed on 7 November 2022)) was created.

In 2014, the national center for diagnostic investigations of stranded marine mammals (Centro di referenza nazionale per le indagini diagnostiche sui mammiferi marini spiaggiati, C.Re.Di.Ma.) was established. This institute cooperates with other entities of the national strandings network that includes the Cetacean stranding emergency response team (CERT), the Mediterranean marine mammals tissue bank of the University of Padua, and the BDS.

In this context, here we gather and report data on fin whale mortality along the Italian coast between 1624 and 2021, highlighting spatial–temporal patterns of distribution of mortality events. Furthermore, by investigating recent mortality events, we evaluate whether significant differences exist in the biological characteristics of the specimens using data spanning 57 years (1964–2021).

## 2. Materials and Methods

### 2.1. Collection of Mortality Data

Information on mortality events was collated through a comprehensive content review of available published scientific literature, grey literature, historical accounts, and available stranding databases and datasets including descriptions of mortality accounts. For the latter, we specifically retrieved data from the BDS (last accessed on 20 September 2022), the Mediterranean Database of Cetacean Strandings (MEDACES—http://medaces.uv.es/; last accessed on 20 September 2022), the database of the Centro Studi Cetacei Onlus (GeoCetus—http://geocetus.it; last accessed on 20 September 2022) [46], and the International Whaling Commission Global Ship Strikes Database (IWC-GSSD—https://iwc.int/management-and-conservation/ship-strikes; last accessed on 21 May 2022). Finally, we accessed reports of CSC Onlus from 1981 to 2010 and the Marine Mammals Sightings database of CIMA Foundation (MAMAS—https://mamas.cimafoundation.org/; last accessed on 20 September 2022).

Museum collections across Italy were also examined to obtain first-hand information on previously unreported mortality events and to cross-check those events with missing details, in particular for older accounts, and/or to resolve discrepancies between sources, where possible. For each event, the following information was collated in full where available: date of the event, number of animals, sex, total body length (TBL), geographic location and coordinates, cause of death, status of the recovered carcass/animal, and other relevant ancillary notes. Whenever possible TBL as available from the original sources were cross-checked with museum collections to correct the estimates. This was carried out by comparing condyle-premaxilla measurement to the TBL of specimens preserved in museum collections [47,48,49,50]. From the date and the TBL we derived the season of the event and the life stage, respectively. Seasons were defined as Spring (SP)—March to May, Summer (S)—June to August, Fall (F)—September to November and Winter (W)—December to February.

Life stages were defined as fetus/calf/newborn (TBL< 10 m), immature (TBL = 10–15 m), sub-adult/adult/mature (TBL > 15 m) [51]. While different morphological features would allow for more accurate identification of life stages and age classes [52], details on these features were not always available for the collated mortality events, in particular for older reports. Accordingly, to avoid bias introduced by potentially skewed data, in this paper we only used TBL to derive individual life stages.

When the exact geographic coordinates of the mortality event were not available in the original sources, they were approximated as accurately as possible based on the event description and available graphical material.

The conservation status of each specimen was classified where possible as alive (Code 1); dead, the specimen died a few hours after sighting/stranding (Code 2); moderate decomposition (Code 3); advanced decomposition (Code 4); mummified/bones (Code 5); and NA (not available/missing) [53].

All events for which discrepancies could not be resolved and, in general, could not be attributed with certainty to fin whales, were discarded. The specimen recently identified as a hybrid between a fin whale and a blue whale [54] was included in the final dataset based on the fact that the proportion of animals sampled for similar investigations across the years is unknown and as a consequence, several other specimens identified as a fin whale could in fact have been a hybrid animal that went unknown. Strandings of live animals that were refloated or reached the open sea (i.e., did not die) were excluded from the dataset. Animals that stranded alive but subsequently died were included with the original date of the stranding.

Wherever possible, the causes of mortality were assessed and cross-checked amongst sources and categorized into natural/biological and human-related. For historical data, each event was classified as stranding (i.e., a category including all animals for which the proximal cause of mortality was not identified), killing, bycatch, ship strike, and floating carcass. For modern data, each event was classified as ship strike, bycatch, and natural/biological (e.g., congenital disease).

The final dataset inclusive of all mortality categories was analyzed to assess spatial–temporal patterns and to evaluate the biological characteristics of the specimens.

### 2.2. Statistical Analysis

#### 2.2.1. Temporal Analysis

Before evaluating patterns in the time series, potential outliers were discarded from the dataset. To assess the temporal evolution of annual mortality events along the Italian coast, we performed structural-change analysis with the aim of highlighting break points in the time series and suggesting changes in the pattern of mortality events over time. Specifically, we used the *strucchange* package [55,56] for the software for statistical analysis R [57] to assess deviations from stability in a classical linear regression model.

First, we used F statistics (Chow test statistics) to highlight the existence of at least one break point in the time series and, therefore, the existence of at least two different time segments. Secondly, we tested the time series for the existence of multiple break points, where each potential break point is estimated alongside its lower and higher confidence intervals [55,56].

For this analysis, when the day of the mortality event was missing, we used the 15th day of the month or, if both the day and month were missing, the date of the 15th of June of the specified year. This analysis was performed on the entire dataset.

#### 2.2.2. Spatial Analysis

To visualize stretches of coastline with higher numbers of mortality events, a 50 km by 50 km grid was overlaid on the Italian coastline, and for each cell of the grid, the number of mortality events was calculated. Each cell was then classified as ‘low’, ‘medium’, or ‘high’ mortality based on the percentile values of the number of mortality events per cell. ‘Low’ was assigned to values lower than the 25th percentile, ‘medium’ to values higher than or equal to the 25th and smaller than the 75th percentile, and ‘high’ to values greater than or equal to the 75th percentile.

#### 2.2.3. Spatial–Temporal Analysis

Space-time cubes (STC) were created in ArcGIS Pro 2.5 (Esri Inc., 2020, Redlands, CA, USA) and were used to visualize and analyze spatial–temporal patterns of the whole dataset of collated mortality events. Before evaluating potential patterns in the time series, outliers were discarded from the dataset.

Data points were aggregated based on a 50 km spatial and 25 yr temporal scale, and the count of points per bin was calculated. Mann-Kendall statistics for each independent location were used to assess whether an increasing or decreasing trend was present in the time series [58]. The result of the test was compared to the expected result of no trend over time to determine if the observed result was statistically significant, based on the Z-score (standard deviations) and *p*-values (statistical probabilities) for each space-time bin, where a small *p*-value (<0.05) indicated that the trend was statistically significant. Hence, the generated STC was analyzed in an Emerging Hot Spot Analysis framework through Getis-Ord (Gi*) statistics [59,60].

The hot and cold spot trends detected by the Getis-Ord Gi* hot spot analysis were evaluated with the Mann-Kendall test to determine whether trends were persistent, increasing, or decreasing over time, by comparing the Z-score of each bin in the STC to neighbouring bins. A Z-score ≥ 1.96 or ≤ 1.96 signifies a statistically significant hot spot or cold spot, respectively, at a significance level of *p* < 0.05. Under this approach, a hot spot is therefore a section of the coastline with statistically significant clustering in both space and time.

The output of the Getis-Ord (Gi*) statistics on the STC was then visualized as a two-dimensional representation uniquely rendered into different categories, describing the statistical significance of hot or cold spots and the trend of the location over time.

#### 2.2.4. Correlation Analysis

The descriptive-observational character of the study orientated the statistical strategy towards a correlative approach to assess the presence of significant correlations between death cause, season, life stage, and other descriptors. Based on the results of the breakpoint analysis, a subsample of modern events was selected to evaluate temporal, spatial, morphometric/biological and event-related descriptors. For each event, time—defined as the number of consecutive days since the first modern record was registered—was log10-transformed, and a time-index expressed as the rank order of time was generated and included in the analysis alongside the variable season. Spatial variables included the latitude and longitude of each event and the region (i.e., the administrative region where each event occurred). Morphometric/biological descriptors included TBL, life stage and sex.

Event-related descriptors were the cause of death, a categorical variable divided into natural/biological, ship strike, and bycatch classes, and cause of death stemming from the former by combining ship strike and bycatch classes into the single ‘human-related’ category. Missing data were reported in a ‘NA’ category for the above descriptors.

One derived categorical variable (cluster) was generated by the application of k-means non-hierarchical clustering to Latitude/Longitude. The clustering was restricted to the Tyrrhenian Sea to avoid potential bias introduced by the clustering procedure based on Euclidean metrics not being able to account for the presence of barriers (e.g., the Italian peninsula between the Tyrrhenian and Adriatic Seas). This variable has three categories corresponding to the Liguria/Tuscany, Sardinia and southern Italy data-driven clusters, respectively.

Correlation between fully numeric variables was computed by the Pearson correlation coefficient, while correlations between categorical variables were estimated by Chi-square metrics.

## 3. Results

Overall, 179 mortality events were found between 1624 and 2021 of which 125 were categorized as ‘stranding/unknown’, 24 as ship strikes, 16 as killing events, 9 as biological/natural (after necropsies) and 5 as bycatch/entanglement events.

Three outlier years were highlighted in the time series of mortality events (1624, 1713 and 1728) (Figure S1 in Supplementary Materials) and therefore were removed from the dataset for further spatial, temporal and spatial–temporal analysis.

### 3.1. Temporal Analysis

**Figure 1:**
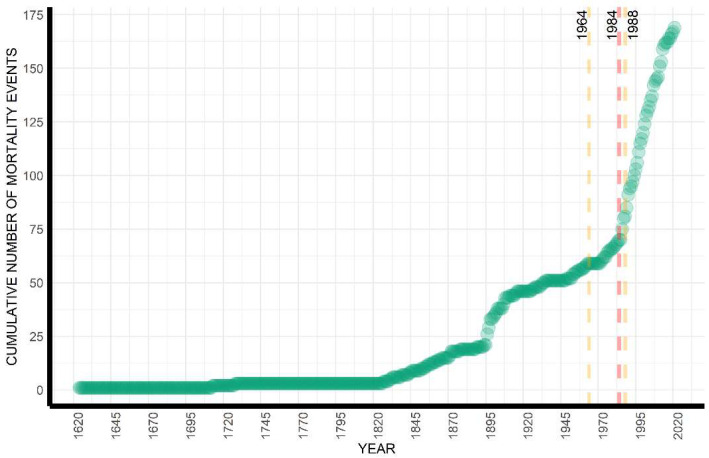
The cumulative number of mortality events between 1624 and 2021. Although the years 1624, 1713, and 1728 were highlighted as outliers in the time series and not included in the formal analysis, they are included here to depict the entire dataset. Dashed red lines represent the breakpoint in the time series and the dashed orange lines represent the breakpoint Lower and Upper Confidence Intervals.

**Figure 2:**
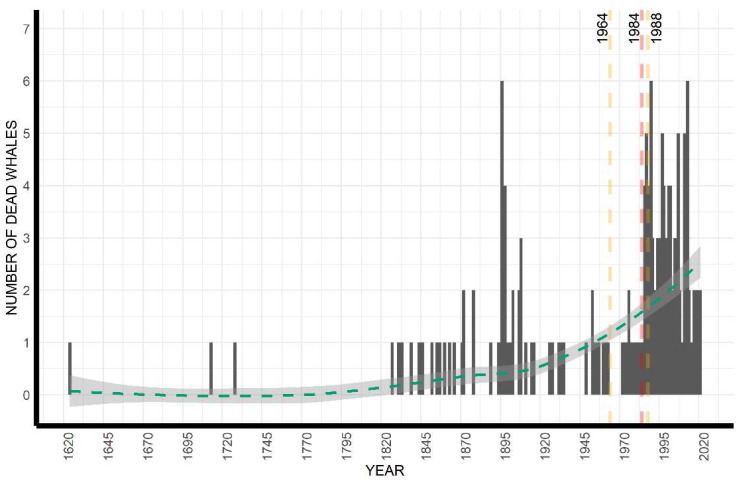
The total number of dead fin whales by year between 1624 and 2021. Although the years 1624, 1713, and 1728 were highlighted as outliers in the time series and not included in the formal analysis, they are shown here to depict the entire dataset. Dashed red lines represent the breakpoint in the time series and the dashed orange lines represent the breakpoint Lower and Upper Confidence Intervals.

The breakpoint analysis highlighted the occurrence of only one structural change in the time series, the year 1984 (LCI = 1964; UCI = 1988), and, consequently, an optimal two-segment partition in the reconstructed time series of mortality events (Figure 1). This breakpoint shows that when looking at the overall evolution of fin whale mortality events over the last five centuries, one significant change in the trend in mortality has occurred, with an increase in the observation of mortality events. The pattern of cumulative mortality events through time (Figure 1) and the pattern of cumulative number of dead animals strongly matches (Figure S2) because most of the mortality events involved a single animal. Figure 2 shows the annual number of dead fin whales between 1624 and 2021 and highlights the relatively higher number of casualties around 1900 and in general in recent years from 1980 onwards (Figure S3 shows the annual number of fin whale mortality events between 1624 and 2021).

Based on the results of the breakpoint analysis, the data from 1964 onward are considered ‘modern’ data and included in the formal Correlation Analyses. Mortality events before 1964 are considered ‘historical’ data. It should be noted that in this study, we used the term stranding, including the definitions of strandling and beaching [53], to improve readability. However, this term only indicates the consequence of death cause but not the proximal cause of mortality, which in most cases remains unknown. Without post-mortem examinations on each specimen, we cannot establish with certainty the cause of death, so it is more appropriate to indicate hypothetical causes of death. Summaries of collated information for these two temporal datasets are provided in Table 1 and Table 2, respectively (for more details on collated information, see the full dataset in the Supplementary Materials).

### 3.2. Spatial Analysis

**Figure 3:**
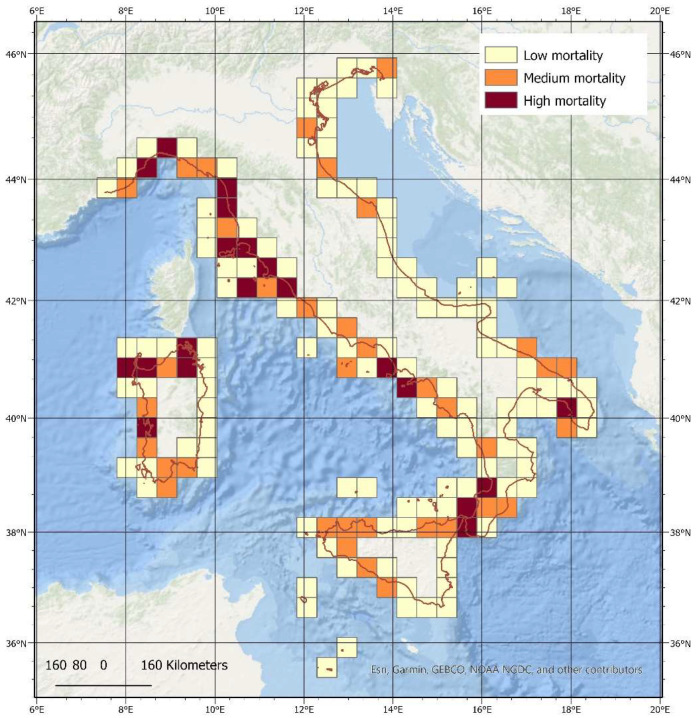
The spatial distribution of fin whale mortality events recorded between 1624 and 2021. Areas with a higher number of mortality events (in dark red) are observed primarily along the coast of the Liguria, Tuscany and northern Sardinia regions and to a lesser extent, along the coast of Campania and in the area of the Strait of Messina. The Italian coastline is highlighted in red.

Most of the mortality events observed in this study occurred in the western sector of the Mediterranean Sea, and this agrees with current knowledge of a strong west-east longitudinal gradient in the occurrence and numbers of fin whales in the Mediterranean region. Sections of the Italian coastline with higher numbers of mortality events are shown in Figure 3. Overall, a higher number of mortality events were recorded for the central and northern coasts of Italy along the Tyrrhenian Sea, along the coast of the Liguria, Tuscany and northern Sardinia regions, along the Coast of Liguria on the Ligurian Sea and, to a lesser extent, along the coast of Campania and in the area of the Strait of Messina in the southern portion of the Tyrrhenian Sea. The Adriatic basin, where the species presence is notably scant, shows relatively few mortality events, in particular along the central and southern coast of Apulia and in the Gulf of Trieste.

### 3.3. Spatial–Temporal Analysis

**Figure 4:**
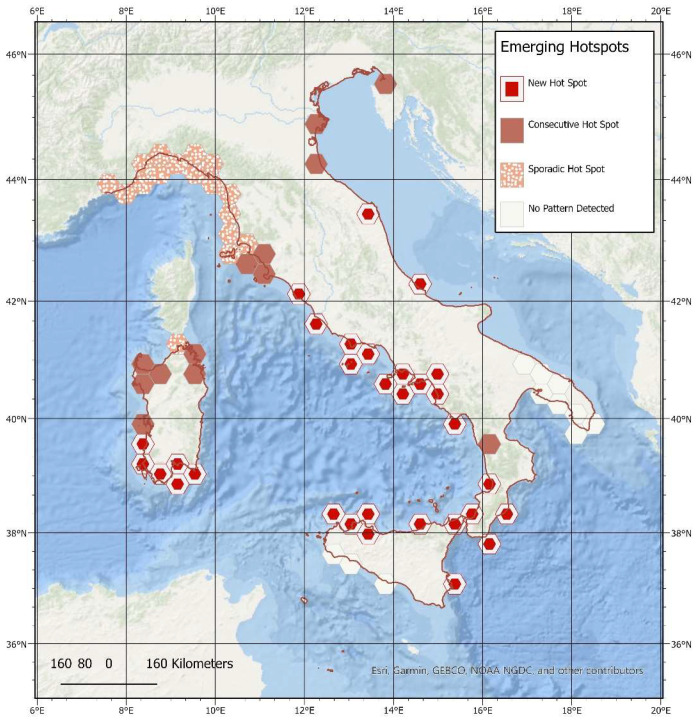
Emerging hot spots obtained from all mortality events from data compiled between the years 1624 and 2021 along the Italian coastline. The Italian coastline is highlighted in red.

The analysis detected four emerging hot spot categories: ‘No pattern’; ‘New hot spot’, defined as an area that has become a statistically significant hot spot in the last few time passages; ‘consecutive hot spot’, defined as an area that was a statistically significant hot spot for a considerable time frame before the final stage; and ‘sporadic hot spot’, defined as an area that was an on-and-off hot spot throughout the entire period. No ‘Cold spots’ or ‘Decreasing cold spots’ emerged from the analysis. Figure 4 shows the above-mentioned areas overlaid with the Italian peninsula. Consecutive hot spots are evident along the coast of Tuscany and in southern Italy along the northern coast of Sicily and the southern coast of Calabria, in the Strait of Messina area, as well as in the southwestern Gulf of Taranto. New hot spots were highlighted along the coast of the Lazio and Campania regions.

### 3.4. Correlation Analysis

**Figure 5:**
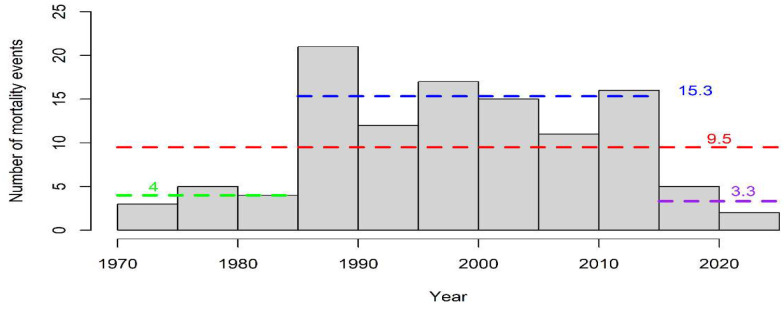
The frequency distribution of modern mortality events over time. Dashed red, blue, green and purple lines show the average number of mortality events over the entire period (1964–2021; the only 1964 record was excluded from the analysis due to lack of information), over the periods 1985–2015, 1970–1985 and 2015–2021, respectively.

The distribution of modern mortality events (Figure 5) highlights two sharp changes at years 1985 and 2015, respectively, while between 1985 and 2015, there is a relatively stable number of mortality events. A Kruskal-Wallis rank sum test suggested a statistically significant difference between the number of mortality events binned at 5-year intervals for the periods 1970–1985, 1985–2015 and 2015–2021 (chi-squared = 8.4824, df = 2, *p*-value = 0.01439). As expected from the distribution of mortality events (Figure 5), statistically significant differences emerged between the periods 1970–1985 and 1985–2015 (two-sample Kolmogorov-Smirnov test: D = 1, *p*-value = 0.02381) and the periods 1985–2015 and 2015–2021 (two-sample Kolmogorov-Smirnov test: D = 1, *p*-value = 0.02381).

As expected, all the temporal variables were highly correlated with Pearson r going from r = 0.65 (*p* < 0.0001) in the case of Index and log time to practical identity (r = 0.99; *p* < 0.0001) for Index and time.

Latitude and Longitude were moderately correlated (r = −0.54, *p* < 0.0001) in accordance with the Northwest to Southeast inclination of the Italian peninsula. The correlation value corresponds to the cosine director of Italy with respect to the north-south meridian axis and thus is an indirect indication of a sufficiently dense coverage of Italian seas by the scored events.

The elevated number of records with ‘NA’ for cause of death (*n* = 77) drastically decreased the statistical power of the Chi-square test in the evaluation of the relation between life stage and death cause (Chi-square = 7.86, *p* = 0.097). However, we observed that the main cause of calf mortality was biological (4 out of 7 corresponding to 57.14% of the class), while ship strikes (14 out of 19) and bycatch (all three recorded events) affected mainly immature animals.

In order to increase the power of the test, we evaluated the correlation between life stages and a simplified biological/not biological binary classification of the cause of death, with the latter reaching a marginal statistical significance: Chi-square= 6.19 (*p* = 0.045), Fisher exact test = 0.040) (Table 3).

A non-statistically significant correlation between death cause (in both multi-class and binary versions) and season was observed; and a lack of statistically significant correlation was observed as well for season and life stage, and for season and region. However, in general, summer showed the lowest number of mortality events, while autumn showed the highest. This trend is particularly evident for the Sardinia region, while the opposite applies to the Liguria region, where the highest number of events occurred during the summer, as already shown by previous studies [33].

**Figure 6:**
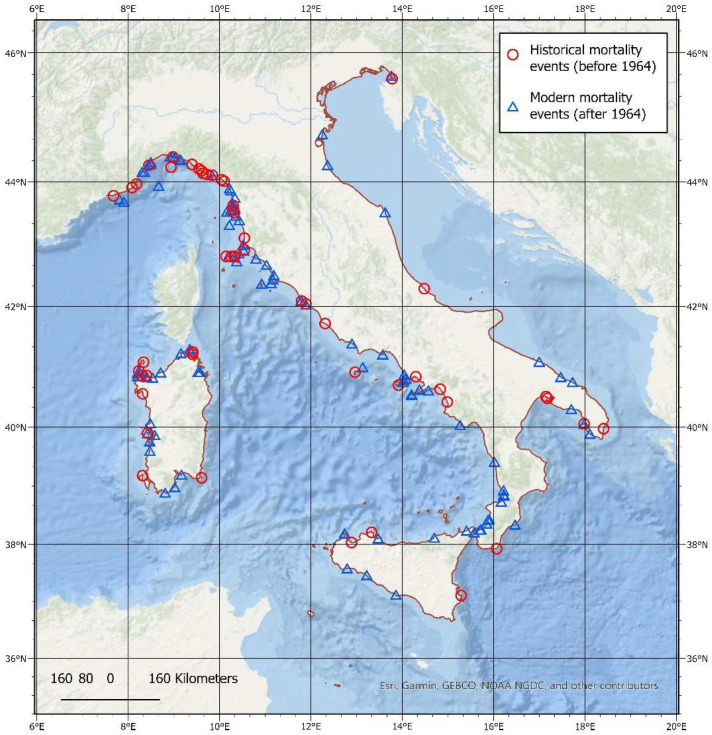
The overall distribution of both historical and modern mortality events along the Italian coast.

The spatial distribution of historical and modern mortality events from 1973 to 2021 is shown in Figure 6. It is evident that mortality events are distributed uniformly along the Italian coast, with the highest occurrence along the western coast of Italy and along the coasts of the islands of Sardinia and Sicily.

**Figure 7:**
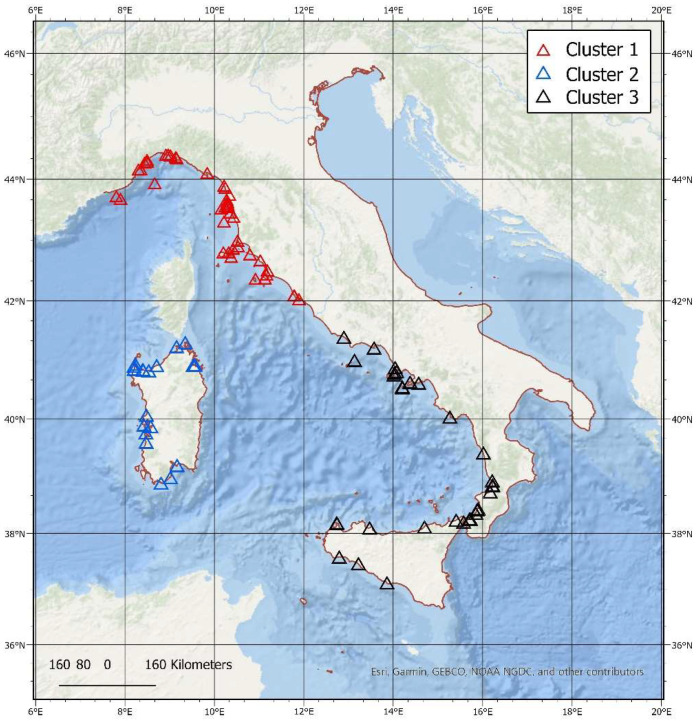
The spatial representation of the three clusters resulting from the clustering procedure. Cluster 1 = Liguria-Tuscany cluster, Cluster 2 = Sardinia cluster and Cluster 3 = southern cluster.

In order to achieve a quantitative representation of the above condition, we performed a cluster analysis by using a non-hierarchical k-means procedure of the spatial location of the events in the Latitude-Longitude space. For a consistent Euclidean space, we limited ourselves to the western basin (Tyrrhenian Sea and Strait of Sicily), which can be considered as unbiased by the spurious underestimation of distances between points separated by land (e.g., Tuscany and Emilia-Romagna locations). The clustering procedure produced an optimal spatial classification into three classes with an R-square equal to 0.84 (to be compared to an R-square = 0.68 in the case of a Gaussian distribution, with a Pseudo-F value = 256.19, *p* < 0.0001). The resulting clusters (Figure 7) are the Liguria-Tuscany (Cluster 1), Sardinia (Cluster 2), and a southern one (Cluster 3).

It is worth noting that two northern Lazio events joined the Liguria-Tuscany cluster and two southern Lazio locations went into the southern cluster.

The clusters did not significantly differ amongst each other either for the life stage or the season of events or death cause, pointing to a common structure of the observed classes.

## 4. Discussion

Here we present a comprehensive dataset of fin whale mortality events that occurred along the Italian coast over four centuries between 1624 and the end of 2021, and an associated geo-referenced database (Supplementary Materials). Such a dataset offers a basis for further investigation into how mortality events of the Mediterranean sub-population of fin whales are affected by a correlation between the effects of oceanic processes on the distribution of specimens and the effects of anthropogenic activity.

The structural change analysis of the time series of mortality events highlights the existence of one single breakpoint—i.e., a single point of significant change in the temporal pattern of mortality—the year 1984 (Cis = 1964–1988). Similar investigations of long-time series events [61] have shown that the sharp increase in mortality of fin whales emerging in the mid-1980s is most likely artificial, caused by a strong contribution of observer bias in reporting. While the reporting of strandings or mortality events in the past was highly biased towards populated and/or easily accessible places, modern national stranding networks tend to distribute their efforts homogeneously across a given area. The breakpoint year 1984 very closely matches the year of establishment of the CSC in 1985 that presented the basis for the first stranding network in Italy and, therefore, the systematic collection of mortality-related data. Additionally, alongside the systematic effort of a dedicated network, the observed increase in events is likely due to an increase in the human population and the generalized development of coastal areas through time [62]. Finally, in Italy, a clear shift in public attitude towards stranded/dead whales emerged in 1970s and 1980s [61], possibly due to public awareness and conservation efforts. This change in attitude, in turn, could have affected the likelihood of reporting a stranding or the presence of a floating carcass.

When considering only modern mortality events over the last six decades (events occurring after 1964), this study shows a regular trend of recorded mortality between 1985 and 2015 with an average of 10 dead animals per year (range = 11–21), and two transition phases, one increasing and one decreasing, before 1985 and after 2015, respectively, with a similar average number of events (4 and 3 animals; ranges = 3–4 and 2–5). The distribution of mortality events binned at five-year intervals for 1985–2016 years (Figure 5) is statistically different from the earlier and later periods. The lower number of mortality events at the beginning of the time series could be explained by the lack of a centralized national stranding network for the systematic collection of information on mortality. The decrease in mortality observed after 2015 seems to be independent of a decrease in anthropogenic-related mortality. Indeed, the last case of bycatch was reported in 1997, while during 2010–2015, when mortality was still relatively high (16 events), only one ship strike was recorded compared to no ship strikes occurring between 2015 and 2020. While the causes of the apparent decline remain unknown, a lack of notifications is unlikely, especially for large cetaceans such as fin whales, in the age of social networks [63]. Ecosystem changes are a plausible hypothesis as well as the influence of climatic variations. Indeed, both large-scale climatic changes [16] and ecosystem changes [64] can explain the periodic variability of cetacean stranding and these aspects need further investigation. Moreover, cetacean strandings can be used as indicators of dynamics of cetacean populations at sea [65] and recent studies have shown that temporal changes in stranding/mortality can be due to changes in cetacean abundance and distribution [65]. Accordingly, a point to consider and that needs further investigation is the potential link between the observed decrease in mortality and the 10% decline of the Mediterranean population of fin whales, inferred by comparing large-scale surveys conducted 27 years apart [24,25,27,32,66,67].

The analysis of modern events suggest little seasonal variation (Figure S4, Supplementary Materials) with a peak recorded in autumn and lowest mortality observed in winter and summer. When considering the sole spatial level, most mortality events occurred along the western coast of Italy and in general across the western Italian seas, mirroring the at-sea distribution of fin whales that is primarily restricted to the western sector of the Mediterranean basin [42,68,69]. The areas with higher recorded mortality numbers are located along the coast of Liguria and Tuscany, Campania and Lazio, north of Sardinia and the area of the Strait of Messina between Sicily and continental Italy. This partially overlaps with current knowledge of fin whale distribution in the western Mediterranean Sea [32,66,70]. While the effect of wind and surface currents on the distribution of carcasses at sea and on shore alike is unknown, we cannot exclude the possibility that the differential distribution of mortality events compared to fine-scale distribution of animals at sea could result from the effects of oceanic and atmospheric processes [6,16,71]. At the same time, this differential distribution could suggest a shift in the at-sea distribution of animals.

When considering spatial–temporal patterns, our analysis highlighted the presence of several recurring hot spots (i.e., consecutive hot spots) along the central coast of Tuscany, in the Gulf of Trieste, north Adriatic Sea and along the northern coast of the island of Sardinia. The recurring presence over time of mortality events in these areas could be due to a regular presence of fin whales in the adjacent waters that could exploit them as seasonal foraging grounds or migratory corridors. Alternatively, specific characteristics in marine circulation patterns, coupled with the geomorphology of the sea floor, could facilitate the beaching of fin whales in these areas. We also noted the emergence of mortality hot spots (i.e., new hot spots), along the entire coast of central and southern Italy, including the northern coast of Sicily and the area of the Strait of Messina as well as the southern coast of Sardinia. These newly established mortality hot spots could be the result of a general shift in the distributional range of fin whales. Fin whales migrating from the southern winter feeding grounds in the Strait of Sicily towards the summer feeding grounds in the waters of the Pelagos Sanctuary navigate through bodies of water with increasing volumes of maritime traffic [22], potentially resulting in higher collision rates and therefore strandings in the southern Italian seas. However, the generalized decrease in mortality associated with ship strike during the last decades suggests that this anthropogenic cause of mortality only marginally contributed to the observed increase in mortality in the southern Italian seas. Furthermore, it cannot be excluded that the spatial–temporal emergence of new hot spot areas of mortality can be a direct consequence of changes in the intensity and location of pressures [65]. Two areas of new hot spots in the time series were also identified along the western-central and northwestern coast of Italy. Fin whales are rarely present in the Adriatic Sea [42,72]. Therefore, we can attribute the position of these modern mortality hot spots to the combined effect of currents, surface winds, and coastal and bottom morphology that push carcasses into this area or direct sick animals or animals with difficulties onto specific areas of the coast, which then strand, dead or alive. Sporadic hot spots were located exclusively along the coast of Liguria and the northern coast of Tuscany. While the Ligurian Sea and more generally the Corso-Liguro-Provençal basin is a well-known seasonal feeding ground for the species [33,42,72,73], the relative paucity of strandings in this area can be a consequence of the characteristics of marine circulation. This study does not show the emergence of any cold spot, persistent or new, along the Italian coastline, which would indicate a decrease in the numbers of recorded mortality events over time and space.

Most of the events were of single individuals, with only two cases of mass stranding involving two animals each, both recorded in the second half of the 1800s. This agrees with the non-gregarious nature of the species, with animals normally present singly, in pairs or in small groups [74,75]. However, large temporary aggregations of fin whales have been reported elsewhere [76]. Unusual mortality events were absent.

Mortality was higher for younger age classes, with mortality of immature individuals predominantly occurring in spring, being 57.14% higher than for adults and other age classes. This result agrees with previous studies highlighting that mortality for Mediterranean fin whales tends to be higher in the earlier stage of life (77%) and to decrease with maturity [77].

It has been suggested that the Mediterranean Sea, compared to the oceans, might have provided year-round resident fin whales an extended and more flexible calendar of breeding and feeding opportunities [42], considering the minor predation pressure and a high potential for socialization due to the relatively small size of the basin. However, increasing pressure from human activity such as vessel traffic, noise, chemical pollution, and likely climate change, raises concern for the population’s survival.

Mortality appears to be in general high for the Mediterranean sub-population of fin whales [42] and this is particularly concerning when considering the recent population size estimates [24]. We did not find evidence of widespread systematic epidemics, compromised immune systems or generalized poor health conditions at the population level over the study period. While full post-mortem examinations were not performed on all recovered specimens between 1964 and 2021, evidence suggests that direct mortality derived from anthropogenic activity is likely to result in a decline of the overall population. When looking at the entire dataset, only four confirmed bycatch events were recorded, three of which occurred in the last six decades with the last one recorded in 1997. Direct killings account for 15 of the recorded events. There was never a commercial whaling tradition within the Mediterranean Sea [61,72], and killing of live-stranded animals or animals in difficulty likely occurred opportunistically rather than being the result of a systematic whaling expedition. The majority of killing events in this study were reported in the second half of the 1800s and the first half of the 1900s, with the last direct killing occurring in 1960 [68]. Additionally, a similar pattern of mortality due to direct killing emerged for another large cetacean inhabiting the Mediterranean Sea, the sperm whale *Physeter macrocephalus* (Linnaeus, 1758) [61]. The sudden stop in this practice, particularly with large cetaceans, could be the result of education and public awareness campaigns, as well as conservation efforts, which in turn have led to a change in attitude towards stranded whales [78]. Amongst the anthropogenic causes of mortality, ship strikes were the most common. The fin whale is the cetacean species that collides with vessels most frequently [38]. The Mediterranean basin, in particular its northwestern sector, has been identified as a collision hot spot for the species [33,34,35,36,37], where it is estimated that between 6% and 20% of Mediterranean fin whales show collision marks [36]. Our results agree with existing knowledge and show that mortality associated with whale–vessel collisions is in general high, and involves younger animals in particular [33]. While the dynamics of whale–vessel collisions are not fully understood, several underlying factors have been identified [79]. Specifically concerning the higher rate of collisions involving younger animals, the fact that they might spend more time at the surface than an adult increases the risk of a collision [80]. Moreover, some authors describe the curiosity of younger animals as a strong contributing factor to increased ship-strike mortality in the earlier life stages [79]. However, the nature of fin whales as a *flight species* [81,82], i.e., a species that tends to swim away from predators or more generally from an actual or perceived threat, provides more support for different hypotheses to explain the higher rate of ship strikes involving younger animals.

Our work shows that in the last decades there has been a putative decrease in whales dying because of a collision. While carcasses of whales struck by vessels might not always be recovered and, therefore, the observed decrease would be merely artificial, it is important to highlight that not only the immediate direct death of a whale following a collision is a matter of concern. Recent studies show that up to 20% of living Mediterranean fin whales show signs of collision [36], with the resulting injury having the potential to affect swimming and diving patterns, leading to altered feeding habits and potentially harmful effects in the long term. While the energetic cost of human disturbance has been assessed in several cases [83,84], similar studies are not yet available for animals that have suffered collisions, and to date, it remains unclear whether the long-term effects of non-lethal collisions on Mediterranean fin whales should be considered a costly unnatural threat rather than a short-term incident. Given the relatively high number of animals that died from collisions in our study, we further stress the need to effectively mitigate the threats posed by ship strikes and to reduce the risk of collision through appropriate management measures, in particular within the framework of the International Maritime Organization.

## 5. Conclusions

Investigations of time series of mortality events can complement existing approaches to gain knowledge on the biology, ecology and conservation status of species, with the benefit of providing a unique source of information for rare and difficult-to-access taxa. It is crucial to highlight that the interpretation of the outcome of these types of studies, in particular concerning the spatial and temporal patterns, must be taken with extreme caution. In the case of cetaceans, the lack of a robust probabilistic sampling of stranding events, associated with different reporting efforts over time, could lead to biased results. However, in this study, the descriptive analytical approach renders the results useful to support informed management and conservation measures. Furthermore, this study strongly supports the importance of a centralized national stranding network as well as the creation and development of detailed datasets on species mortality. At the same time, it also highlights that in Italy, despite four decades of systematic collection of mortality data, often the proximal causes of mortality remain unknown. It thus emphasizes the need to develop integrated and standardized data collection procedures which allow the assessment of the health status of the Mediterranean sub-population of fin whales.

Existing knowledge suggests that current mortality rates for Mediterranean fin whales might be unsustainable, in particular in light of the recent abundance estimates across the basin. Direct mortality associated with ship strikes remains high, and there is a lack of information on the potential detrimental long-term population effects of non-lethal whale–vessel collisions. Alongside ship strikes, the combined effects of high levels of chemical pollution [39,85], the ingestion of high volumes of microplastics [86,87,88], and the impact of underwater noise pollution [43] further stress the necessity of developing an overarching conservation and management plan (CMP) for the species in the Mediterranean Sea. In this context, the International Whaling Commission and the Agreement on the Conservation of Cetaceans of the Black Sea, Mediterranean Sea and contiguous Atlantic area are in the process of developing this much-needed CMP with the overall aim of managing human activity afflicting Mediterranean fin whales and supporting their favorable conservation status across the entire range of distribution within the region. Our study provides relevant input to the development of this CMP.

## Figures and Tables

**Table 1:** Summary of the main variables of historical mortality events (1624–1963). N/A Not available, (*) Here, the cause of death is known.

Variable	Description	Value	%
No. of animals		67	
Sex	Male	12	17.91
	Female	10	14.92
	Unknown	45	67.16
Life stage	Fetus/Calf/Newborn	8	11.94
	Immature	16	23.88
	Sub-adult/Adult/Mature	29	43.28
	NA	14	20.89
Total length (m)	Minimum	5.50	
	Maximum	21.50	
Cause of death	Hypothetical	19	28.35
	Unknown	48	71.64
Hypothetical human-related cause of death	Killing *	16	84.21
	Bycatch	2	10.52
	Ship strike	1	5.26
Season	Autumn	16	23.88
	Winter	16	
	NA	14	20.89
	Summer	14	
	Spring	7	10.44
Region	Liguria	17	25.37
	Tuscany	15	22.38
	Sardinia	10	14.92
	Lazio	7	10.44
	Campania	5	7.46
	Puglia	5	
	Sicily	3	4.47
	Calabria	2	2.98
	Friuli-Venezia Giulia	2	
	Abruzzo	1	1.49
Carcass/Body conservation status	Alive (Code 1)/dead (Code 2)	2	2.98
	NA	65	97.01

**Table 2:** Summary of the main variables of modern mortality events (1964–2021). NA Not available, (*) Estimated and measured lengths, (°) One animal was Code 5 (Mummified/Bones).

Variable	Description	Value	%
No. of animals		112	
Sex	Male	27	24.10
	Female	38	33.92
	Unknown	47	41.96
Life stage	Fetus/Calf/Newborn	32	28.57
	Immature	47	41.96
	Sub-adult/Adult/Mature	24	21.42
	NA	9	8.03
Total length (m) *	Minimum	2.70	
	Maximum	19.77	
Cause of death	Hypothetical	35	31.25
	Unknown	77	68.75
Hypothetical cause of death	Human-related	26	74.28
	Natural/Biological	9	25.71
Human related cause of death	Ship strike	23	88.46
	Bycatch/entanglement	3	11.53
Season	Autumn	34	30.35
	Spring	28	25.00
	Summer	25	22.32
	Winter	25	
Region	Tuscany	28	25.00
	Sardinia	20	17.85
	Liguria	18	16.07
	Calabria	11	9.82
	Campania	9	8.03
	Sicily	9	
	Puglia	6	5.35
	Lazio	5	4.46
	Emilia-Romagna	2	1.78
	Friuli-Venezia Giulia	1	0.89
	Marche	1	
	Uncertain location	2	1.78
Carcass/Body conservation (°)	Advanced decomp. (Code 4)	54	48.21
	NA	34	30.35
	Fresh (Code 2)	15	13.39
	Alive (Code 1)/dead (Code 2)	8	7.14

**Table 3:** Relative contingency table showing the expected increase in natural/biological causes of death for calves and a higher number of human related causes of death for immature animals.

Death Cause/Life Stage	Sub-Adult/Adult/Mature	Immature	Fetus/Calf/Newborn
Natural/Biological	3	2	4
Human related	6	17	3

## Data Availability

Collected data used for the analyses in this study are provided as Supplementary Materials to this paper.

## Supplementary Materials

The following is available online at https://www.mdpi.com/article/10.3390/ani12223111/s1, Table S1: Dataset 1624–2021 (Excel file), Figure S1: Boxplot for the years with mortality events considered in this study and highlighting the three outliers years 1624, 1713 and 1728; Figure S2: The cumulative number of dead fin whales between 1624 and 2021. Although the years 1624, 1713, and 1728 were highlighted as outliers in the time series and not included in the formal analysis, they are included here to depict the entire dataset. Dashed red lines represent the breakpoint in the time series and the dashed orange lines represent the breakpoint Lower and Upper Confidence Intervals; Figure S3: The total number of mortality events by year between 1624 and 2021. Although the years 1624, 1713, and 1728 were highlighted as outliers in the time series and not included in the formal analysis, they are shown here to depict the entire dataset. Dashed red lines represent the breakpoint in the time series and the dashed orange lines represent the breakpoint Lower and Upper Confidence Intervals; Figure S4: Seasonal distribution of modern (1964–2021) fin whale mortality events.
