# Supplementary material for: Fin Whale (Balaenoptera physalus) Mortality along the Italian Coast between 1624 and 2021
Source: Animals (Basel). 2022 Nov 10;12(22):3111. doi: 10.3390/ani12223111 (PMC9686696; doi:10.3390/ani12223111)
Supplement: Supplementary file 1 [file animals-12-03111-s001.zip › Figures S1-S4.pdf]

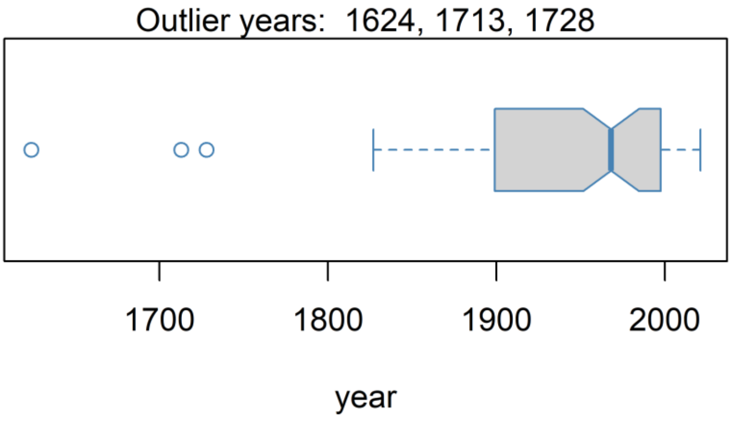

Figure S1. Boxplot for the years with mortality events considered in this study and highlighting the three outliers years 1624, 1713.and 1728.

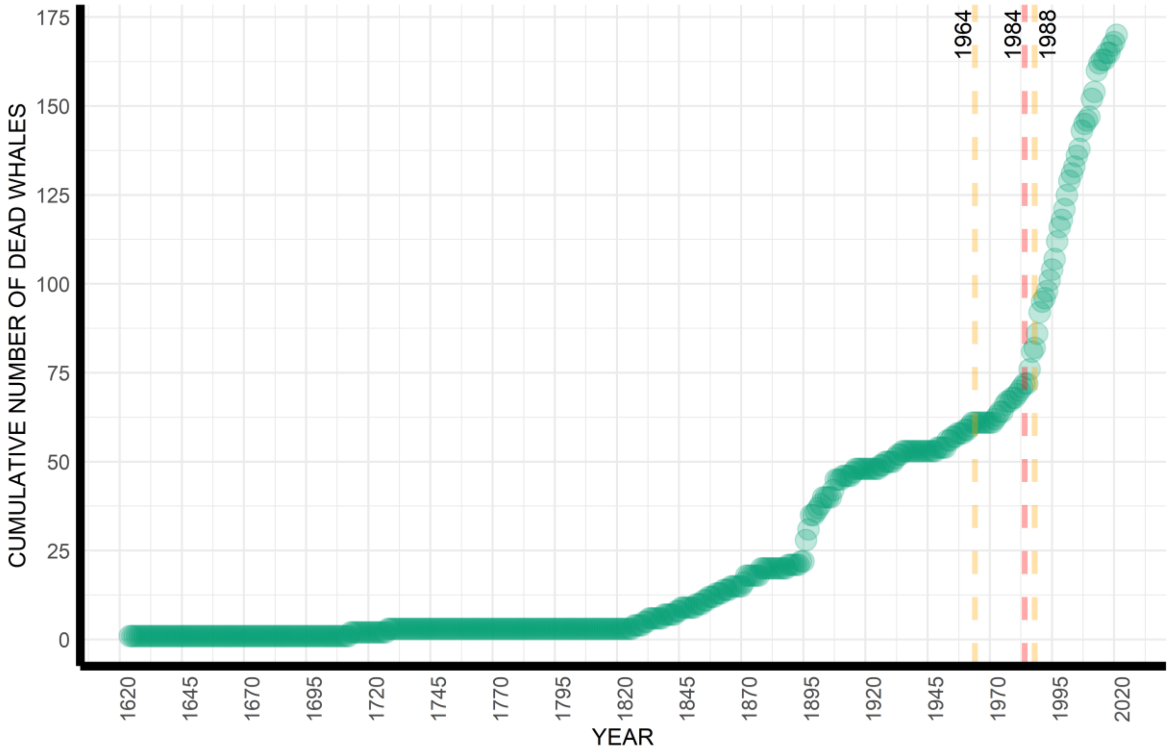

Figure S2: The cumulative number of dead fin whales between 1624 and 2021. Although the years 1624, 1713, and 1728 were highlighted as outliers in the time series and not included in the formal analysis, they are included here to depict the entire dataset. Dashed red lines represent the breakpoint in the time series and the dashed orange lines represent the breakpoint Lower and Upper Confidence Intervals.

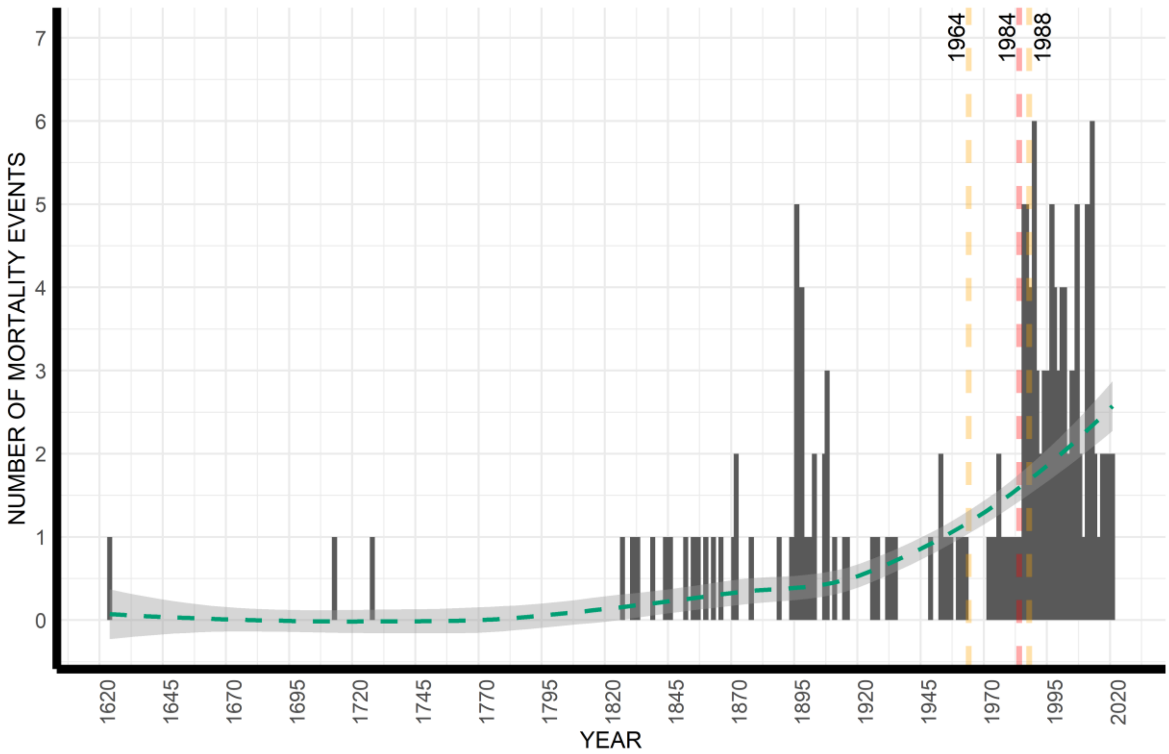

Figure S3: The total number of mortality events by year between 1624 and 2021. Although the years 1624, 1713, and 1728 were highlighted as outliers in the time series and not included in the formal analysis, they are shown here to depict the entire dataset. Dashed red lines represent the breakpoint in the time series and the dashed orange lines represent the breakpoint Lower and Upper Confidence Intervals.

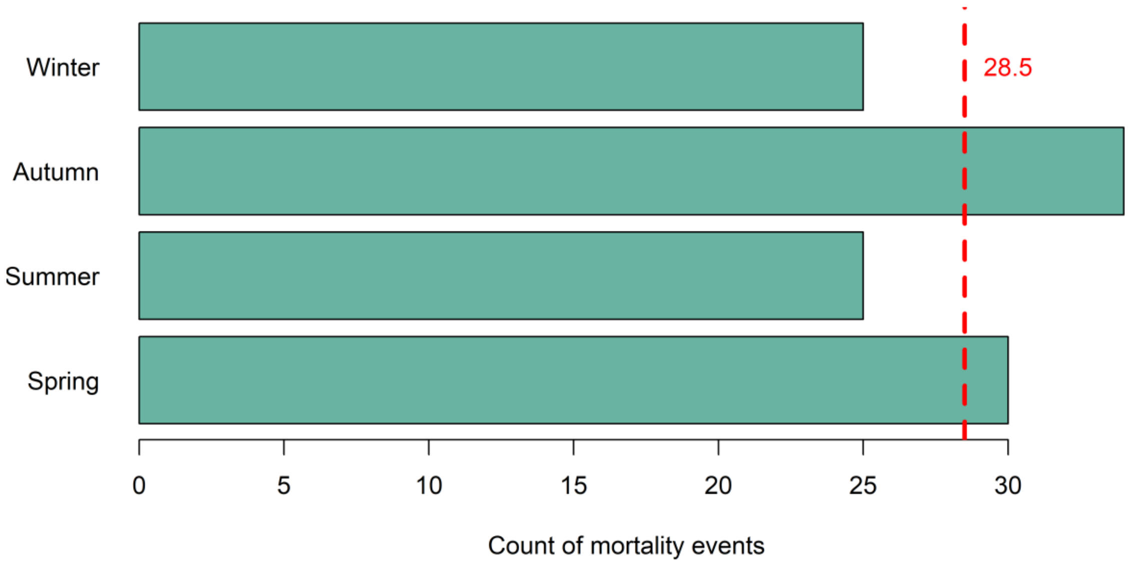

Figure S4: Seasonal distribution of modern (1964–2021) fin whale mortality events.
